# Role of Nephronectin in Pathophysiology of Silicosis

**DOI:** 10.3390/ijms20102581

**Published:** 2019-05-26

**Authors:** Suni Lee, Machiko Honda, Shoko Yamamoto, Naoko Kumagai-Takei, Kei Yoshitome, Yasumitsu Nishimura, Nagisa Sada, Shigeyuki Kon, Takemi Otsuki

**Affiliations:** 1 Department of Hygiene, Kawasaki Medical School, 577 Matsushima, Kurashiki, Okayama 701-0192, Japan; slee@med.kawasaki-m.ac.jp (S.L.); s.yamamoto@med.kawasaki-m.ac.jp (S.Y.); kumagai@med.kawasaki-m.ac.jp (N.K.-T.); kei_y@med.kawasaki-m.ac.jp (K.Y.); yas@med.kawasaki-m.ac.jp (Y.N.); nagisada@okayama-u.ac.jp (N.S.); 2 Department of Pharmacy and Pharmaceutical Sciences, Fukuyama University, Gakuen-cho 1, Fukuyama, Hiroshima, Hiroshima 729-0292, Japan; honda@fukuyama-u.ac.jp (M.H.); kon@fukuyama-u.ac.jp (S.K.); 3 Department of Biophysical Chemistry, Graduate School of Medicine, Dentistry and Pharmaceutical Sciences, Okayama University, Okayama 700-8530, Japan

**Keywords:** silicosis, extracellular-matrix, nephronectin, serum concentration, factor analysis

## Abstract

Silicosis is a typical form of pneumoconiosis and is characterized as a type of lung fibrosis. Silica particles are captured and recognized upon by alveolar macrophages via the macrophage receptor with collagenous structure (MARCO) scavenger receptor, and thereafter the inflammasome is activated. Thereafter, various chemokines/cytokines play their roles to eventually form fibrosis. Additionally, silica particles chronically activate T helper cells which sets the background for the formation of silicosis-associated autoimmune disturbances. The occurrence and progression of lung fibrosis, the extracellular matrix-related molecules such as integrins and their ligands including fibronectin, vitronectin, laminin, and collagens, all play important roles. Here, the roles of these molecules in silicosis-related lung fibrosis are reviewed from the literature. Additionally, the measurement of serum nephronectin (Npnt), a new member of the integrin family of ligands, is discussed, together with investigations attempting to delineate the role of Npnt in silica-induced lung fibrosis. Serum Npnt was found to be higher in silicosis patients compared to healthy volunteers and seems to play a role in the progression of fibrosis with other cytokines. Therefore, serum Npnt levels may be employed as a suitable marker to monitor the progression of fibrosis in silicosis patients.

## 1. Introduction

Silicosis is a well-known and typical pneumoconiosis caused by silica inhalation [1,2]. Inhaled silica particles are initially recognized by alveolar macrophages [3,4]. These alveolar macrophages function as dendritic cells that recognize foreign danger signals such as silica particles and asbestos fibers [5,6]. The first contact between these cells and silica particles is importantly mediated by macrophage receptor with collagenous structure (MARCO) scavenger receptors [7,8]. The study by Thakur et al. using MARCO knock-out versus wild-type mice revealed MARCO-mediated enhancement of acute inflammation and lung injury as well as chronic inflammatory reactions [9,10]. Moreover, Chen reported that blocking of interleukin (IL)-17 led to silica-induced lung inflammation and fibrosis delay in a mouse model [10]. Additionally, this group showed that Th17 cells regulate silica-induced lung inflammation via an IL-1β-dependent manner [11]. Furthermore, this group found that blocking the Wnt/β-catenin pathway suppressed regulatory T (Treg) cell skewing, and enhanced the Th17 response. This block then also caused enhancement of silica-induced lung inflammation [12]. They also showed inhibition of the Th17 response by Baicalin, a flavone glycoside, naturally found in several species of the genus Scutellaria and acts as prolyl endopeptidase inhibitor. Other groups showed the importance of MyD88, the adaptor molecule in Toll-like receptor, and IL-1 receptor signaling in formation of silica-induced inflammation and formation of fibrosis [13]. Taken together, it seems that various immune circumstances surrounding silica particles and alveolar macrophages may influence the occurrence of silica-induced lung inflammation and development of lung fibrosis known as silicosis.

After capturing silica particles by alveolar macrophages, the process of activation of the inflammasome proceeds [5,6,14,15]. The nucleotide-binding oligomerization domain receptor, or NOD-like receptor (NLR), pyrin domain-containing 3 (NLRP3) inflammasome belonging to the NLR subfamily of pattern-recognition receptors is activated to perform its function. The inflammasome is a multi-protein complex comprising the NLRP3 protein itself, the adapter molecule designated as ASC (Apoptosis-associated Speck-like protein containing a caspase activation and recruitment domain (CARD)), and caspase-1 [5,6,14,15]. Following activation of this protein-complex, pro-inflammatory cytokines IL-1β and IL-18 are converted into their active forms by caspase-1. Thereafter, these cytokines summon fibroblasts to form accumulated collagen fibers as well as initiate the inflammatory cascade including pro-inflammatory cytokines such as IL-6, transforming growth factor (TGF)-β and tumor necrosis factor (TNF)-α [4,16,17,18,19].

Additionally, various chemokines such as Monocyte Chemotactic Protein-1 (MCP-1, also known as chemokine (C-C motif) ligand 2 (CCL2)), Macrophage Inflammatory Proteins (MIP) α (known as CCL3) and β (CCL4) are involved to form silica-induced lung inflammation and fibrosis to activate and recruit various inflammatory cells such as neutrophils, macrophages, and monocytes [20,21]. Additional cells are summoned including various lymphocytes such as CD4+ helper T cells (effector T (Teff) cells) and differentiated subtypes such as Th1 as well as Treg cells and Th17 cells. The relatively balanced population of these Th subtypes may also contribute to form continuous chronic inflammation [22,23].

Moreover, the balance of Th subtypes may contribute to the basic immunological dysregulation of autoimmune tolerance [15,24]. Patients with silicosis are often complicated with various autoimmune disorders such as rheumatoid arthritis (known as Caplan’s syndrome), systemic sclerosis and anti-neutrophil cytoplasmic antibody (ANCA)-induced vasculitis/nephritis [25,26,27]. Regarding immune alterations caused by silica exposure, our investigations have revealed that recurrent and chronic exposure to silica particles induces chronic activation of Teff and Treg cells [28]. Activated Teff cells yield various markers for activation such as CD69 and program death (PD) -1 molecules on their surface and secret soluble IL-2 receptor (sIL-2R) [29]. Additionally, these Teff cells produce inhibitory molecules against Fas-mediated apoptosis such as soluble Fas (sFas), including other alternatively-spliced Fas variants. These molecules compete with the binding site for Fas ligand but lose membrane-binding domains like typical sFas, and decoy receptor 3 (DcR3) molecule against TNF-related apoptosis-inducing ligand (TRAIL) signaling. Thus, Teff cells in silicosis become chronically activated in addition to surviving longer by preventing Fas-mediated apoptosis. On the other hand, Treg is also chronically activated, whereas these activated Treg cells express significantly more Fas molecules on their cell surface, resulting in earlier loss of Treg cells [24,25,26,27,28,29,30].

In this article, involvement of extracellular matrix components such as the integrin family in the progression of silica-induce lung fibrosis is reviewed. Additionally, the role of ligands for the integrin family such as fibronectin, vitronectin, laminin, and collagen are also summarized. Furthermore, although no reports have been published to date concerning silicosis and nephronectin (Npnt), another member of the integrin ligand, our group investigated the serum levels of Npnt in silicosis and studied factors related to Npnt among various cytokines and respiratory factors.

## 2. Silicosis and Integrin

The integrins play an important role in terms of the cell extracellular matrix (ECM). Following ligand binding, activated integrins lead to initiation of signal transduction pathways for cell cycle progression, organization of the intracellular cytoskeleton, and presentation of various cell membrane receptors. Integrins form heterodimers [31,32]. They possess two subunits designated α and β. There are many α and β subunits in human cells. All subunits penetrate the cell membrane and possess several cytoplasmic domains. The main function of integrins is attachment of the cell to the ECM and signal transduction from the ECM to the cells. Furthermore, integrins also affect immune surveillance, cell migration, and the binding between some pathogens such as viruses and cells. Fibronectin, vitronectin, collagens, and laminin are known to be ligands for integrin [33,34]. Npnt, which is involved in kidney development, is also a novel ligand for integrin. Npnt plays other roles such as in cell adhesion, differentiation and spreading [35,36,37].

Regarding Npnt regulation, this integrin was initially found to act with integrin α8β1 during kidney development [34]. The high-affinity binding between Npnt and integrin α8β1 was confirmed to involve the Arg-Gly-Asp motif. [36]. More recently, it was reported that Npnt is a potential prognostic marker in breast cancer and that Npnt mediates p38 mitogen-activated protein kinase (MAPK)–induced cell viability [38]. Npnt expression was suppressed by fibroblast growth factor (FGF)-2 via c-Jun N-terminal kinase (JNK) in MAPK and Phosphoinositide 3-kinase (PI3K) pathways [39]. However, no correlations have been reported in the case of lung fibrosis.

Several investigations have been performed in an effort to delineate the role of integrin in the onset of silicosis. For example, Bodo et al. investigated ECM metabolism and viability of human bronchial epithelia cells with respect to different sizes and shapes of silica particles [40]. In that study, they found that commercially obtained silica samples promoted stronger expression of the integrin β5 gene as well as fibronectin, Matrix metalloproteinase (MMP)-12 and tenascin genes compared to silica particles used for casting gold jewelry [40]. Then, they suggested that silica particles may affect ECM production via metalloproteases and TGF-β signal pathways to form lung fibrotic changes. Nardi et al. investigated inflammatory and oxidative stress parameters in silicosis. They measured various parameters including β-2 integrin, L-selectin and intercellular adhesion molecule (ICAM)-1 expression on the cell surface of lymphocytes and monocytes as well as other serum parameters in silicosis patients [41]. Although β-2 integrin was not extracted as a biomarker for silicosis progression, they found L-selectin expression would be an early biomarker in silica-exposed workers. More recently, Yuan et al. reported that micro RNA (miR)-542-5p inhibited the proliferation and migration of NIH-3T3 fibroblast treated with TGF-β1 [42]. Then the target of miR-542-5p was integrinα6. Additionally, the knockdown of this integrin caused phosphorylation of the [Focal adhesion kinase (FAK) / PI3K/ protein kinase B (PKB, known as AKT)] pathway. Thus, they suggested the importance of Integrin α6 in the development of silica-induced lung fibrosis and that its inhibition (by miR-542-5p) may be a potential therapeutic target for the treatment of silicosis [42].

Taken together, results indicate that the integrin family may affect the development of lung fibrosis caused by silica exposure.

## 3. Silicosis and Ligands for Integrin Family

### 3.1. Fibronectin (FN)

From the classical studies reported from the 1980s to 1990s, results indicated that FN increased in lung tissues and bronchoalveolar lavage fluids (BALF) of silicosis patients [43,44,45,46]. More recently, several reports have appeared that detail inhibiting the progression of silica-induced lung fibrosis using animal models. For example, neutralization of IL-1β, bone morphogenetic protein (BMP)-7 and bone marrow mesenchymal stem cells (BMSCs) were challenged for this reason. In all of these studies, mRNA expression or histochemical staining of FN were used to monitor the degree of silica-induced lung fibrosis [47].

Since FN is known to play important roles in cell adhesion, growth, migration, and differentiation, the critical roles of FN in fibrosis in various organs includes the lungs as well as in cancer cell biology [48,49].

### 3.2. Vitronectin

Vitronectin is also a ligand for integrin. Less work has been reported regarding the role of this ligand in silicosis. One study showed that vitronectin protected alveolar macrophages from silica toxicity [50]. This study showed that vitronectin levels increased in BALF of silica-treated rats. Furthermore, it was shown that vitronectin possessed affinity for silica and that, using a 51Cr release assay, it was shown that the presence of vitronectin protected silica-induced injury of cultured alveolar macrophage by reducing free radical production. In another study, Zetterberg et al. investigated the comparison of alveolar and interstitial macrophages in the fibrosing stage following quartz exposure [51]. Their studies found that adhesion of interstitial macrophages to vitronectin increased following exposure, whereas exposed alveolar macrophages adhered to a lesser extent to vitronectin and FN.

Although it is difficult to assess the role of vitronectin in silicosis from these reports, it may play some role in the development of silica-induced lung fibrosis.

### 3.3. Laminin

Laminin is also a ligand for integrin. Some relatively old reports have been published regarding observations of laminin in silicosis. Haustein reported silica-induced scleroderma/systemic sclerosis (SSc) [52]. In that report, silicosis patients complicated with SSc showed increased levels of collagen metabolism markers including laminin peptide-P1 in addition to other makers such as β-galactosidase and N-terminal procollagen type III peptide. Herrmann et al. also reported that SSc patients with silicosis showed increased levels of laminin PI fragment in their serum [53]. Segade et al. reported that RAW 264.7 mouse macrophages treated with silica showed increased gene expression of 67-kDa high-affinity laminin receptor and other genes. Additionally, Bodo et al. compared the silica powder used in casting gold jewelry and commercially available silica with respect to fibrogenic effects on epithelial bronchial cells [54]. They found that commercially available silica caused down-regulation of laminin with enhanced FGF-2 receptor expression, thus highlighting the importance of the FGF2 signaling pathway in silica exposure.

However, little is known regarding the role of laminin in silicosis.

### 3.4. Collagen

Of course, collagen is produced by fibroblasts stimulated by activated alveolar macrophages following silica exposure, and collagen plays an important role in fibrosis and nodular lesions characterized as silicosis [16,55]. Collagen deposition has been monitored using various experimental silicosis models in an effort to examine various molecular targets for the inhibition of progressive fibrosis caused by silica exposure, such as N-acetyl-seryl-asoarthyl-lysyl-proline (Ac-SDKP; anti-fibrotic tetrapeptide) [56,57], BMSCs (as mentioned above) [58], pirfenidone (specific drug for idiopathic pulmonary fibrosis), and nicorandil (an antianginal and potassium channel opener agent) [59,60].

The pathological findings of silicosis clearly show that the deposition of collagen and progression of fibrosis is related to the interaction between integrins and various ligands including collagen. Preventing the onset of lung fibrosis as well as the progression of fibrosis in silica-exposed workers are very important, especially in developing nations.

**Figure 1:**
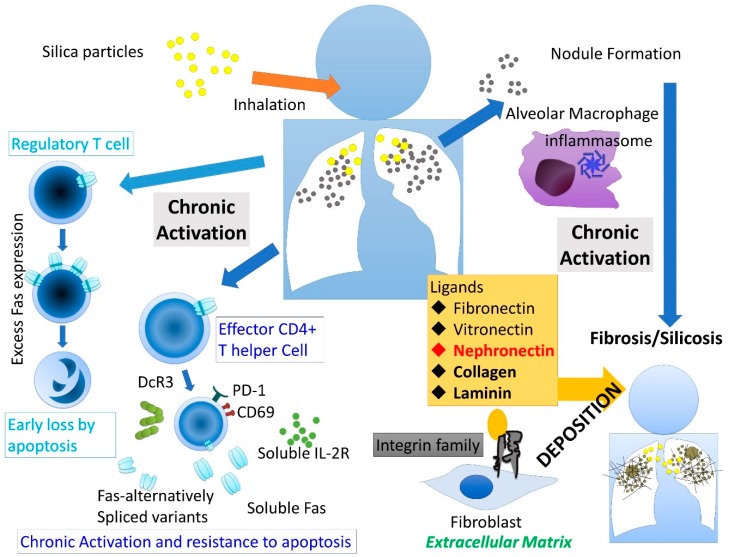
Schematic presentation of the occurrence and progression of silica-induced fibrosis (silicosis) via extracellular matrix including integrins and their ligands such as fibronectin, vitronectin, nephronectin, collagen, and laminin. Additionally, on the left panel, activation of T helper cells by silica exposure is summarized. With respect to effector T cells, silica-induced chronic activation was revealed by expression of PD-1, CD69, and production of soluble IL-2 receptor (sIL-2R), and resistance to apoptosis was demonstrated by excess production of soluble Fas, other alternatively-spliced variants of the Fas gene and over-production of decoy receptor 3 (DcR3). On the other hand, with respect to regulatory T cells, chronic activation induced excess expression of Fas molecules on its surface resulting in early loss by apoptosis.

A summary of the immune alterations and effects on ECM caused by silica exposure is schematically shown in Figure 1.

## 4. Npnt in Silicosis

As mentioned above, the role of Npnt in silicosis has not been reported. Thus, we attempted to examine the role of Npnt in silicosis using serum obtained from silicosis patients. Additionally, although some ligands for integrin have been reported as mentioned above, the roles of Npnt in silica-induced fibrosis as well as in the lung environment remain unclear.

### 4.1. Patients and Healthy Volunteers

All subjects were Japanese. Twenty silicosis patients (SIL) (age: average ± standard deviation (SD) = 74.9 ± 5.4, 19 males and 1 female) and 19 healthy volunteers (HV) (age 44.8 ± 8.6, 9 males and 10 females) were included in this study. All SIL were medically followed at Kusaka Hospital, Bizen City, Okayama Prefecture, Japan, and were employed at the brickyard works. The amount of free silica inhaled by the SIL was supposed to be as high as 40–60%, as determined from examination of their work environment. Bizen city is located on the eastern side of Okayama Prefecture, the west side of Japan, approximately at the midpoint between Osaka and Hiroshima. One of the key industries of Bizen city is a firebrick factory. Many factories were founded approximately 100 years ago and continue their activities to the present. Although work environments and conditions have been improved, prior to the economic growth period of Japan in the 1960s and 1970s, many workers who had inhaled silica dust have since been diagnosed with pneumoconiosis. All SIL were diagnosed with pneumoconiosis according to the International Labor Office (ILO) 2011 revised guidelines [61]. They showed no clinical symptoms of autoimmune diseases such as sclerotic skin, Raynaud’s phenomenon, facial erythema, or arthralgia. Peripheral blood was drawn from the cubital vein from all subjects. All specimens were taken only when informed consent had been obtained. This study was approved by the Ethics Committee of Kawasaki Medical School and Kusaka Hospita (Project number #2719, approved on 24 May 2017). The project follows the rules of the Declaration of Helsinki.

The limitation of this study comprised the small number of SIL patients and HV. Additionally, SIL patients were not well characterized, especially with respect to smoking status and other factors regarding the development of lung fibrosis. Additionally, when collecting samples from HV, it was difficult to collect samples from elderly HV. This is why relatively young HV were enlisted for this study. In an effort to extend the studies regarding immune alterations in SIL patients as well as identifying factors regarding silica-induced lung fibrosis, we are now attempting to collect data from a larger number of SIL patients and correspondingly matched HV.

### 4.2. Measurement of Serum Npnt and Other Cytokines

The ELISA method for detecting Npnt had been developed by Kon et al., originally in the Department of Pharmacy and Pharmaceutical Sciences, Fukuyama University. Twenty-nine kinds of cytokines were measured mainly using a Luminex Human Cytokine and Chemokine 29-Plex Kit (Merck Millipore, Billerica, MA) that included epidermal growth factor (EGF), granulocyte colony stimulating factor (G-CSF), granulocyte-macrophage-colony stimulating factor (GM-CSF), interferon (IFN)-α2, IFN-γ, IL-1α, IL-1β, IL-1ra, IL-2, IL-3, IL-4, IL-5, IL-6, IL-7, IL-8, IL-10, IL-12 (p40), IL-12 (p70), IL-13, IL-15, IL-17A, IP-10 (CXCL10), MCP-1, monocyte chemotactic protein -1 (MIP-1) α, macrophage inflammatory protein (MIP)-1β, TNF-α, TNF-β, vascular endothelial growth factor (VEGF), and Eotaxin/CCL1. Other factors including soluble IL-6 receptor (sIL-6R), TGF-β1 and soluble Fas (sFAS) were measured separately using individual ELISA kits provided by Creative Diagnostics., New York, NY, RayBiotech Inc., Norcross, GA., and MyBioSource.com., San Diego, CA, respectively. Additionally, if individual levels of each cytokine in silicosis patients were less than the lower limits of measurement, one tenth of the lower limit was substituted in lieu of using “nd” as “not detected”.

Details concerning respiratory factors such as age, profusion rate (PR; according to the ILO pneumoconiosis radiological classification, 2011 revised guidelines, 1 to 4), exposure years (Exp.Year; according to occupational history), subjective dyspnea (numbered 1 (slight) to 4 (severe), according to the Hugh–Jones classification), percentage of forced expiratory volume in 1 second (%FEV1), peak flow rate (PFR) at 25% forced vital capacity (FVC)/Height (v25H), and percent forced volume capacity (FVC) were provided by Kusaka Hospital at the time of sampling the blood samples.

### 4.3. Statistical Analyses

All statistical analyses were performed using SPSS ver. 21 (Japan IBM Co. Ltd., Tokyo, Japan). In particular, for factor analysis of the 20 silicosis patients various respiratory, inflammatory, and some growth factor items were chosen from all of the measured items and comprised the following: Respiratory factors: Years of exposure, PR, %VC, and %FEV1]; Inflammatory and Growth factors; IL-1α, IL-1β, IL-2, IL-4, IL-8, IL-10, IL-17, IFNα2, IFNγ, IP-10/CXCL10, MCP-1/CCL2, MIP1α/CCL3, TNFα, and TGFβ; in addition to Npnt.

### 4.4. Serum Npnt in Silicosis Patients and Healthy Volunteers

**Figure 2:**
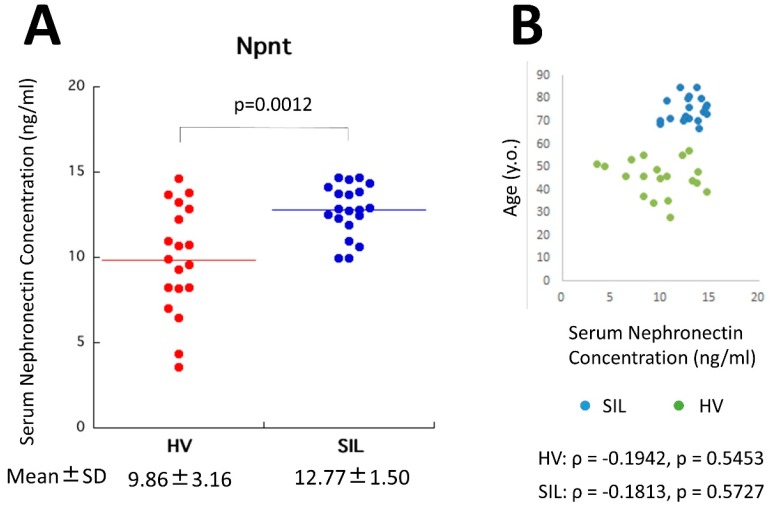
Panel (**A**) shows a comparison of serum nephronectin (Npnt) between healthy volunteers (HV) and silicosis patients (SIL). SIL showed significant higher values of serum Npnt concentrations compared to HV. Panel (**B**) shows the distribution of age and Npnt concentrations in HV and SIL, since HV and SIL showed differences in age. However, in HV, there was no correlation of age and Npnt. Thus, it was assumed the higher Npnt concentrations were unrelated to age.

Figure 2A shows a comparison of serum Npnt levels (ng/mL) between SIL and HV. The Npnt levels were significantly higher in SIL (mean ± standard deviation (SD): 12.77 ± 1.50) compared to HV (9.86 ± 3.16). The only report describing serum Npnt levels was that by Watany and El-Horany [62]. Although their values differed from this report, our ELISA was developed first. Thus, a comparison between SIL patients and HV in our system is discussed here. The only concern was a difference in age between SIL and HV. However, as shown in Figure 2B, Npnt levels in HV were not dependent on age. Among the various ages in HV, the range of Npnt was variable. Although a comparison of Npnt between age-matched SIL and HV will be needed, to date, it is expected that SIL showed higher Npnt levels in their serum.

### 4.5. Correlation between Npnt and Other Cytokines, Growth Factors, and Respiratory Factors.

**Figure 3:**
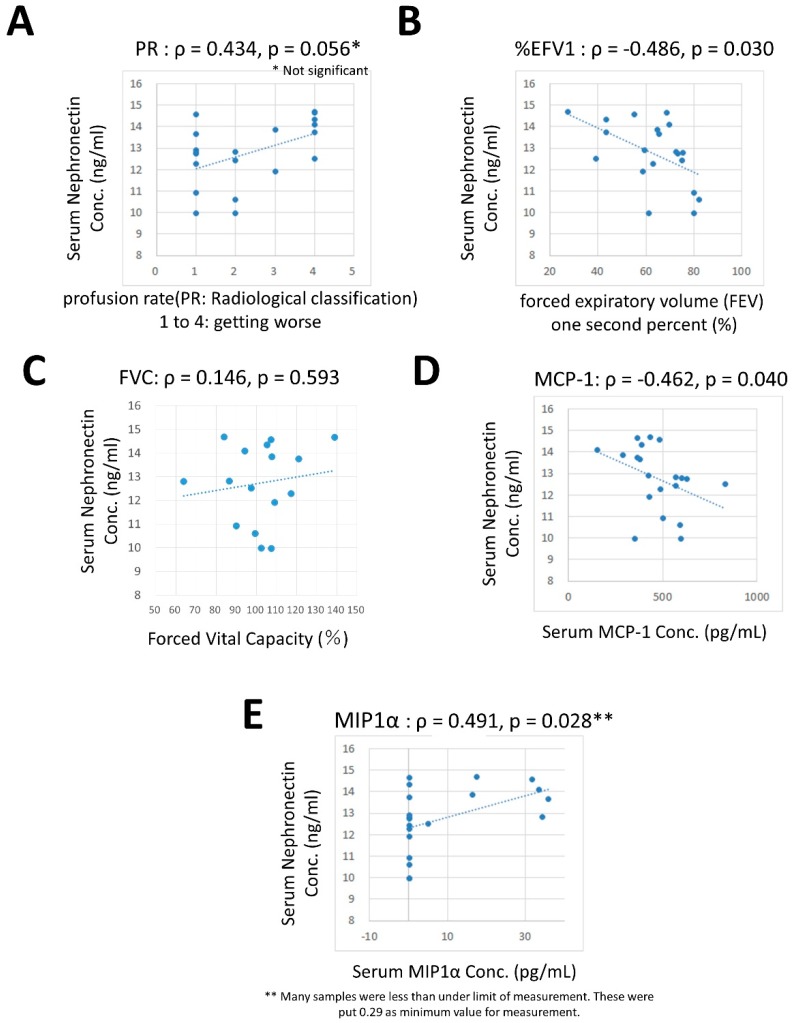
In silicosis individuals, the correlation of Npnt with other respiratory, inflammatory, and fibrosis-related factors were analyzed. Representative relations are shown in Panels (**A**) (PR), (**B**) (%FEV1), (**C**) (FVC), (**D**) (MCP-1), and (**E**) (MIP-1α). Significant correlations were found in Panels (**B**,**D**,**E**) and PR showed tendency towards a positive correlation (Panel **A**). There was no significant correlation between Npnt and FVC. The other factors did not show any significant correlations.

The correlation between serum levels of Npnt in SIL and other factors in silicosis were examined. Significant correlations were found with %FEV1 (inverse, ρ = −0.486 and *p* = 0.030, Figure 3B) and MCP-1 (inverse with ρ = −0.462 and *p* = 0.040, Figure 3D) and MIP-1α (positive with ρ = 0.491 and *p*= 0.028, Figure 3E). Additionally, numbered PR tended to show a positive correlation (ρ = 0.434 and *p* = 0.056, Figure 3A). However, there was no correlation between serum levels of Npnt and FVC. Based on these results, the Npnt levels may be related to a worsening of lung fibrosis (from the PR and %FEV1 values).

Additionally, Npnt levels seemed to be related to inflammatory activity given the MIP-1α results, whereas it should be that Npnt shows an inverse correlation with MCP-1. Furthermore, MCP-1, known as CCL2, acts to recruit monocytes, memory T cells, and dendritic cells to the lesion of inflammation produced by either tissue injury or infection [63,64]. On the other hand, MIP-1α, also known as CCL3, is involved in the acute inflammatory state in the recruitment and activation of granulocytes [65,66]. Thus, both CCLs are mainly involved in the acute phase of inflammation. To consider the roles of both CCLs in lung fibrosis caused by silica exposure, MCP1/CCL2 may not be required at this late phase of forming fibrosis. However, MIP-1α/CCL3 may play a role in contributing to acute inflammation at the border of silicotic nodules. With respect to MIP-1α, half of the SIL showed the lower limit of measurement. Thus, SIL could conceivably be divided into two groups, one group comprises SIL without elevated MIP-1α and who do not possess acute inflammation, while the other group comprises SIL with higher MIP-1α and who may still possess active inflammatory lesions in their pulmonary lesions.

Although our series of MIP1α levels included approximately half of the SIL who showed the lower limit of measurement, examination of MIP1 α revealed a positive correlation with various other cytokines measured in this study. In particular, SIL with higher MIP-1α showed higher levels of IL-3, IL-4, IL-5, IL-7, IL-8, IL-12p40, IL-13, IL-15, IFN-α2, EGF, and VEGF. However, MCP-1 did not show this tendency.

### 4.6. Factor Analysis

Factor analysis represents one statistical approach that can be utilized to find unexpected variables or parameters correlated to various observed variables or parameters. Thus, we utilized this method in an effort to identify the relationship between certain parameters in SIL such as Npnt values with other factors such as inflammatory and respiratory items.

To confirm the aforementioned tendency, factor analysis [67,68] was performed using selected parameters assayed in this study (inflammatory and growth factors, and respiratory factors) as mentioned above. As shown in Table 1, MIP-1α formed factor 1 with IL-8, IP-10/CXCL10, IL-4, IFN-α2, IL-10, IL-1α, and PR. However, MCP-1 was not involved. All these items showed a positive relation. This might be interrupted as inflammatory factor.

Factor 2 shown in Table 1 comprised IL-17, IFN-γ, IL-2, and IL-1α. With IL-17 considered to be strongest in this factor, IL-17 might be the factor related to silicosis-induced disturbance of autoimmunity.

Factor 3 was also of interest, since this comprised MCP-1 as the strongest, with TNF-α as a positive item and MIP-1α and Npnt as negative items. From factor 3, increases in MCP-1 and MIP1α tended to display opposite tendencies. Additionally, Npnt was a negative concern with respect to formation of factor 3. Taken together, MCP-1 and MIP-1α seem to play roles in different pathophysiologies of silicosis. This made the correlation between Npnt and MCP-1 an inverse relationship while that of Npnt and MIP-1α was positive.

Taken together, the role of chemokines and their ligands in silicosis may reveal novel pathological findings regarding the progression and inflammatory status of silicotic lung fibrosis. If the involvement of certain critical molecules can be identified, this could lead to the development of preventive and/or inhibitory molecular targets that should assist silica-exposed populations in the world.

### 4.7. Factors Related to Npnt Levels

**Figure 4:**
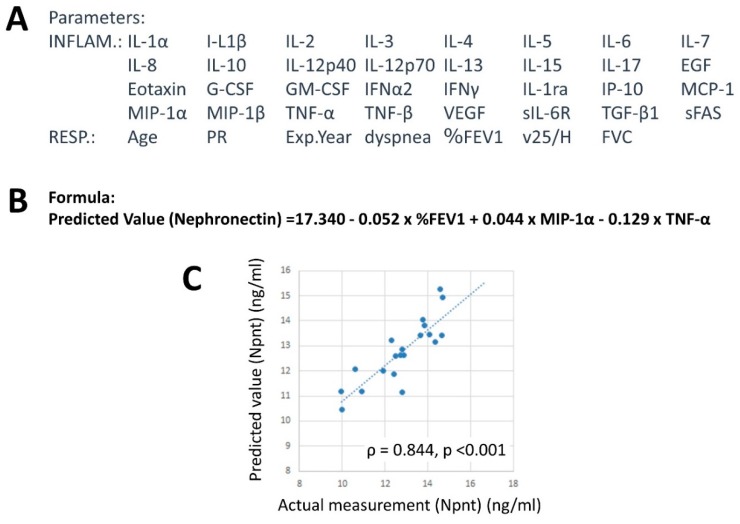
To confirm the role of Npnt in silicosis, multiple regression analysis was employed to identify other parameters among those shown in Panel (**A**) (INFLAM indicates cytokines, chemokines and growth factors measured and RESP indicates parameters related to respiratory pathology). From the results shown in Panel (**B**), the predicted value was demonstrated with formula “Predicted nephronectin (Npnt) = 17.340 − 0.052 × %FEV1 + 0.044 × MIP-1α − 0.129 × TNF-α. In Panel (**C**), the correlation with actual measurement of Npnt and predicted value calculated by the Panel B formula is shown. This showed a strong positive and significant correlation. Thus, %FEV1, MIP-1α and TNF-α are assumed to be related to Npnt levels.

In an effort to identify factors related to the Npnt level, multiple regression analysis was performed. As shown in Figure 4A, parameters related to inflammatory (INFLAM) and respiratory (RESP) factors were included. Thereafter, the following formula was extracted:

Npnt = 17.340 − 0.052 × %FEV1 + 0.044 × MIP-1α − 0.129 × TNFα.

These three values for individual SIL were substituted into this formula and predicted values of Npnt were determined.

As shown in Figure 4C, actual Npnt and predicted Npnt calculated using the aforementioned formula showed significant positive correlation. This result indicated lower %FEV1, higher MIP-1α, and lower TNF-α were involved. The interpretation of these results might be that: (i) Npnt levels are associated with worsening fibrosis since lower %FEV1 resulted and (ii) inflammation, represented as higher MIP-1α, is related to Npnt levels, in whereby Npnt may be associated with inflammatory responses in silicosis. However, the role of TNF-α, which was extracted to generate the formula, was not easily understood as Npnt and TNF-α levels did not reveal a significant correlation (ρ = 0.259 and *p* = 0.271). Contrarily, MCP-1, which showed an inverse correlation as shown in Figure 3C, was not chosen to predict Npnt levels. Of course, there was no correlation between MCP-1 and TNFα. Considering the role of TNFα in the relationship between silicotic lung fibrosis and Npnt levels, TNF-α is known to be involved in initial inflammatory responses that form silicotic nodules and in the recognition of silica particles by alveolar macrophages. However, TNF-α may not be involved in the progression of sequential fibrosis. On the other hand, Npnt may play a role in this later phase to extend the fibrotic and inflammatory responses with MIP-1α and other cytokines related to MIP-1α. Since this is just speculation, further studies involving larger SIL numbers as well as time-sequential sampling are required to unequivocally identify the role of Npnt in silicosis progression.

### 4.8. Summary of NPNT in Silicosis

To summarize the role of Npnt in silicosis, it was shown that Npnt levels are higher in SIL. This indicates that Npnt plays a role in the initiation and progression of silica-induced lung fibrosis. It was apparent that the deterioration of lung function (%FEV1) was associated with higher Npnt levels. Additionally, a worsening of inflammatory changes related to higher MIP-1α was also related to Npnt. Thus, factors related to Npnt pathophysiologically and clinically may be candidate biomarkers and target molecules for the prevention and inhibition of silica-induced lung fibrosis.

However, it has been indicated that profibrotic growth factors such as FGF-2 [39] and TGF-β [69,70] and the profibrotic JAK/STAT pathway [70] suppress the expression of Npnt. These investigations focused on osteogenesis. According to our findings resulting from factor analysis and multi-regression assays, Npnt seemed to be correlated with the late phase of lung fibrosis as well as bronchial alterations, but was not related to initial inflammatory responses affected by TNF-α. Unfortunately, FGF2 levels were not measured in this study, and TGF-β was not related to Npnt levels. Thus, further studies are required to determine the role of Npnt in initial fibrotic and inflammatory changes caused by silica particles, and investigation of chronic and progressive development of silica-induced lung fibrosis should be performed to distinguish the roles of Npnt in separate phases of cellular and molecular events in silicosis.

If Npnt is related to the occurrence and development of lung fibrosis, future studies should involve an examination of serum Npnt levels in other cases of lung fibrosis caused by different occupational substances such as asbestos, indium, or cadmium, as well as idiopathic lung fibrosis. Moreover, a comparison with well documented markers for fibrosis such as KL-6 and surfactant protein–D (SP-D) should also be performed in future studies.

Additionally, the fact that %FEV1, but not FVC, was extracted as a factor related to serum Npnt in SIL patients was slight paradoxical, since lug fibrosis such as silicosis showed restrictive lung diseases detected by decreasing FVC. Usually, bronchial asthma and chronic obstructive pulmonary diseases (COPD) show a reduction in %FEV1. In fact, most SIL patients have complications such as secondary bronchitis and more than half of SIL patients showed a disturbance in %FEV1. This indicated that SIL patients showed mixed, obstructive and restrictive lung status. Therefore, these phenomena may have affected our results. It would be better to monitor Npnt and other markers including cytokines as well as values of lung function tests over time. However, to collect data comprising newly diagnosed SIL patients in Japan is difficult since new cases of SIL usually number less than 100 per year. Thus, larger samples should be collected with various lung disturbance levels to confirm the present results regarding Npnt and %FEV1.

## 5. Conclusions

In this article, the role of ECM, integrin, and their ligands were summarized. Additionally, our study discussed the role serum nephronectin (Npnt), a newly identified ligand of integrin, in silica-induced inflammation and fibrosis. These investigations have only just begun and further research is required to increase our understanding of the role of Npnt as well as other ECM related molecules in silicosis, which will eventually lead to the development of biomarkers, preventive measures, and identification of suitable inhibitory targets that can be utilized to cease the progression of fibrosis. The beneficiaries of this research will be the many people who have been exposed to silica particles in addition to those who continue to be exposed.

## Figures and Tables

**Table 1:** Factor analysis.

	Extracted Factors		
Item	Factor 1	Factor 2	Factor 3
Exp. Year	0.424	−0.040	0.392
PR	−0.145	0.125	−0.099
FVC	0.111	−0.051	−0.236
%FEV1	−0.063	−0.158	−0.126
IL-1α	0.478	0.635	0.136
IL-1β	−0.159	0.012	−0.055
IL-2	−0.134	0.669	−0.091
IL-4	0.791	0.047	0.032
IL-8	0.929	−0.047	−0.276
IL-10	0.552	−0.235	0.237
IL-17	−0.064	1.003	−0.074
IFN-α2	0.717	0.124	0.029
IFN-γ	−0.056	0.968	−0.029
IP-10/CXCL10	0.929	−0.098	0.192
MCP-1/CCL2	0.129	−0.025	1.010
MIP-1α	0.643	0.057	−0.526
TNFα	0.128	0.067	0.469
TGFβ	0.047	−0.288	−0.202
Npnt	0.132	0.089	−0.456
Contribution (%)	17.973	17.736	15.192

Other factors extracted showed less than 10% contribution. Hence, those factors did not seem to be important. Shaded boxes indicate items significantly related to form factors (absolute values are >0.4).

## Abbreviations

MARCO	macrophage receptor with collagenous structure
IL	interleukin
Treg	regulatory T
NLR	NOD-like receptor
NLRP3	pyrin domain-containing 3
ASC	Apoptosis-associated Speck-like protein containing a CARD
CARD	caspase activation and recruitment domain
TGF	transforming growth factor
TNF	tumor necrosis factor
MCP-1	Monocyte Chemotactic Protein-1
CCL2	chemokine (C-C motif) ligand 2
MIP	Macrophage Inflammatory Proteins
Teff	effector T
ANCA	anti-neutrophil cytoplasmic antibody
PD	program death
sIL-2R	soluble IL-2 receptor
sFas	soluble Fas
DcR3	decoy receptor 3
TRAIL	TNF-related apoptosis-inducing ligand
ECM	extracellular matrix
Npnt	Nephronectin
MMP	Matrix metalloproteinase
ICAM	intercellular adhesion molecule
miR	micro RNA
FAK	Focal adhesion kinase
PI3K	Phosphoinositide 3-kinase
PKB	protein kinase B
FN	Fibronectin
BALF	bronchoalveolar lavage fluids
BNP	bone morphogenetic protein
BMSCs	bone marrow mesenchymal stem cells
SSc	systemic sclerosis
FGF	fibroblast growth factor
Ac-SDKP	*N*-acetyl-seryl-asoarthyl-lysyl-proline
SIL	silicosis patients
SD	standard deviation
HV	healthy volunteers
ILO	International Labor Office
EGF	epidermal growth factor
G-CSF	granulocyte colony stimulating factor
GM-CSF	granulocyte-macrophage-colony stimulating factor
IFN	Interferon
VEGF	vascular endothelial growth factor
sIL-6R	soluble IL-6 receptor
PR	profusion rate
Exp.Year	exposure years
%FEV1	forced expiratory volume in 1 second
PFR	peak flow rate
FVC	percent forced vital capacity
v25H	25% forced vital capacity (FVC)/Height
FVC	percent volume capacity
INFLAM	inflammatory
RESP	Respiratory
JAK	Janus kinases
STAT	signal transducer and activator of transcription
SP-D	surfactant protein–D
